# The association of serum prolidase activity with developmental dysplasia of the hip

**DOI:** 10.1007/s00296-013-2672-9

**Published:** 2013-01-22

**Authors:** N. Soran, O. Altindag, N. Aksoy, H. Çakır, A. Taşkın, M. Soran, E. Işıkan

**Affiliations:** 1 Department of Physical Medicine and Rehabilitation, Research Hospital, Harran University, Sanliurfa, Turkey; 2 Department of Biochemistry, Research Hospital, Harran University, Sanliurfa, Turkey; 3 Department of Pediatry, Research Hospital, Harran University, Sanliurfa, Turkey; 4 Orthopaedy and Traumatology, Research Hospital, Harran University, Sanliurfa, Turkey; 5 Department of Physical Medicine and Rehabilitation, Research Hospital, Gaziantep University, Gaziantep, Turkey

**Keywords:** Prolidase, Developmental dysplasia of the hip, Collagen turnover

## Abstract

The purpose of this study was to evaluate serum prolidase activity in patients with developmental dysplasia of the hip (DDH). Prolidase enzyme activity was measured spectrophotometrically to pointing out the collagen metabolism. The prolidase activity in patients with DDH was significantly higher than that in the control group (*p* = 0.002). Furthermore, there was positive correlation between prolidase activity and dysplasia level. Increased serum prolidase activity may have played a role in the presence of DDH. We therefore hypothesized that the increased prolidase activity related to collagen turnover may be associated with etiopathogenesis and/or the progression of the disease.

## Introduction

Developmental dysplasia of the hip (DDH) is a common congenital abnormality that affects the developing hip joint of the newborn. DDH is a comprehensive term used to describe a spectrum of anatomical abnormalities of the hip that may be congenital or may develop during infancy and/or childhood. DDH refers to dysplasia of the acetabulum (deficient development) or instability of the hip, such that the femoral head can be partially or completely moved out of the acetabulum [1].

The etiology of hip dysplasia is not clear, but this condition does appear to be related to a number of different factors. Female sex, being the first-born child and breech positioning are all associated with an increased prevalence of DDH. Other factors possibly related to DDH include intrauterine positioning and sex, and some of these are interrelated.

Anatomical abnormalities of the hip joint arose from a deviation in normal hip to the developmental hip disorders including partial or complete displacement of the femoral head from acetabulum during infantile growth period [2]. It has been thought that changes in the connective tissue of joint capsule and ligaments might be responsible for the increased laxity, although this association is less clear.

The hereditary disorders of connective tissues encompass a spectrum of conditions linked pathophysiologically by abnormalities of collagen, fibrillin and matrix proteins [3, 4]. The last step of collagen degradation is mediated by prolidase, which is a cytosolic exopeptidase that cleaves iminodipeptides containing carboxyterminal proline or hydroxyproline, and plays an important role in collagen metabolism [5, 6]. The enzyme also recycles proline from iminodipeptides derived from degradation products of collagen and other proline-containing proteins. This recycling is important for collagen re-synthesis and cell growth [7]. The relationship between collagen and prolidase activity was observed during fibrotic processes, where an increase in prolidase activity was accompanied by increase in tissue collagen deposition. In view of the above facts, prolidase activity may be an important factor in the regulation of collagen biosynthesis. Collagen is essential for the maintenance of connective tissue [8, 9]. In various studies, the aim was to associate collagen metabolism with pathogenesis of the diseases of interest [10–13]. However, to the best of our knowledge, there is no study in the medical literature which is investigated prolidase activity pointing out the collagen metabolism in DDH.

## Materials and methods

### Subjects

The study was conducted at the Harran University Medical Faculty, Departments of orthopedic surgery and Clinical Biochemistry during the years 2007 and 2008. Parents of all patients signed informed consent forms approved the study. Thirty-six patients (29 girls and 7 boys) were included; three patients were bilaterally affected. Thirty-three controls (22 girls and 11 boys) were included. A total of 69 subjects between 9 and 18 months of age were included. All patients were diagnosed as having typical DDH.

Our patients had not undergone any operation before the study. Cartilage metabolism may be influenced by the operation. Secondary hip dislocations owing to neurological pathologies were excluded from this study.

We used Tonnis’s [14] identification criteria for levels of dysplasia of the hips, whereas radiological results according to the Severin [15] scoring criteria. The control groups were free of any medication for at least 4 weeks prior to blood sampling. We performed all patients’ routine hematological and biochemical parameters to exclude any possible systemic disorder which may affect the parameters used in the study.

### Samples

Fasting blood samples were withdrawn and their sera were separated with centrifugation at 3,000 rpm for 10 min, and the serum samples were stored at −80 °C until analysis.

### Measurement of prolidase enzyme activity

Prolidase activity was determined by a photometric method based on the measurement of proline levels produced by prolidase [16–18]. The samples (100 μL) were mixed with 100 μL of serum physiological. A total of 25 μL of the mixture was preincubated with 75 μL of the preincubation solution (50 mmol/L Tris–HCl buffer pH 7.0 containing 1 mmol/L GSH, 50 mmol/L MnCl_2_) at 37 °C for 30 min. The reaction mixture, which contained 144 mmol/L gly-pro, pH 7.8 (100 μL), was incubated with 100 μL of preincubated sample at 37 °C for 5 min. To stop the incubation reaction, 1 mL glacial acetic acid was added. After adding 300 μL Tris–HCl buffer, pH 7.8 and 1 mL ninhydrin solution (3 g/dL ninhydrin was melted in 0.5 mol/L orthophosphoric acid), the mixture was incubated at 90 °C for 20 min and cooled with ice. Absorbance was then measured at a 515 nm wavelength in order to determine proline by the method proposed by Myara et al. [19, 20], which is a modification of Chinard’s method [21]. Intra- and inter-assay coefficient of variations (CV) of the assay were lower than 10 %.

### Statistical analysis

All analyses were conducted using SPSS 11.5 (SPSS for Windows 11.5, Chicago, IL). Continuous variables are expressed as mean ± SD. The normality of distributions was evaluated with the one-sample Kolmogorov–Smirnov test. Student’s *t* test was used for group comparison. A two-tailed *p* < 0.05 was considered statistically significant. Spearman’s correlation analysis was used to determine magnitude of correlations between prolidase level and Tonnis scores.

## Results

The demographic and clinical data of the subjects are summarized in Table 1. The average age were 12.5 ± 2.4 and 14 ± 2.4 months in patients and controls, respectively. There were 29 girls and 7 boys in patients group and 22 girls and 11 boys in healthy controls. There were no statistically significant differences between the two groups with regard to age and sex distribution.

**Table 1:** Demographic and clinical data of the patients and controls

	Patients	Controls	*p*
Case	36	33	>0.05
Age (month)	12.5 ± 2.4	14 ± 2.4	>0.05
Sex (female/male)	29/7	22/11	>0.05
Prolidase (U/L)	575.11 ± 22.66 IU	519.24 ± 9.99 IU	0.001

Data are mean ± SD; >0.05 = non-significant

The mean value of prolidase enzyme activity level was 575.11 ± 22.66 and 519.24 ± 9.99 IU in patients and controls, respectively. The prolidase level was significantly higher in DDH patients than that in control group (*p* < 0.001) (Table 1). In addition, there was positive correlation between prolidase activity and Tonnis scores in patients with DDH (*r* = 0.44, *p* < 0.001) (Table 2).

**Table 2:** Association between prolidase level and Tonnis scores

	Tonnis scores
Prolidase (U/L)	*r* = 0.44, *p* < 0.001

## Discussion

The aim of our study was to associate collagen metabolism with pathogenesis of the diseases of interest. We discovered an interesting relationship between prolidase activity and hip dysplasia. Increased prolidase activity may shed light on the etiopathogenesis DDH.

Despite the intensive research, etiopathogenesis of DDH is still poorly understood. Anatomical abnormalities of the hip joint arose from a deviation in normal hip to the developmental hip disorders including partial or complete displacement of the femoral head from acetabulum during infantile growth period [22]. Although in most affected infants the problem resolves spontaneously in the first several months of life, persisted DDH may result in chronic pain and gait abnormalities [23]. In a typical synovial joint, the ends of opposing bones are covered with a thin layer of articular cartilage. Cartilage clearly performs a mechanical function. It provides a bearing surface with low friction and wear, and because of its compliance, it helps to distribute the loads between opposing bones in a synovial joint. The normal growth of the acetabulum depends on normal epiphyseal growth of the triradiate cartilage and on the three ossification centers located within the acetabular portion of the pubis (os acetabulum), ilium (acetabular epiphysis), and ischium [24].

Normal development of the hip joint requires appropriate alignment and contact between the ball of the femoral head and the socket of the acetabulum. In persistent DDH, the anatomical relationship between the femoral head and the acetabulum is incorrect, leading to abnormal development. In severe cases, a misplaced femoral head leads to the development of a false acetabulum in the pelvis. The relative influence of the collagen network and proteoglycans on the tensile behavior of cartilage depends on the rate of loading. When pulled at a slow rate, the collagen network alone is responsible for the tensile strength and stiffness of cartilage. At high rates of loading, interaction between the collagen and proteoglycans is responsible for the tensile behavior; proteoglycans restrain the rotation of the collagen fibers when the tissue is loaded rapidly. A delicate balance exists between the growing proximal femur, the acetabular and tri-radiate cartilages and adjacent bones that allow the acetabulum to develop. It is not fully understood what controls this balance [24, 25] and what factors are responsible for the degree of development of a false acetabulum.

Abnormality in the shape of the acetabulum and femoral head causes gait abnormalities. In DDH, the acetabulum is too shallow and the head of the femur is insufficient; in addition, there may be generalized joint laxity [23, 25]. It is thought that the laxity of the capsule is a major contributory factor for DDH [26].

Biochemical studies of DDH capsules have been suggested that the amount of type III collagen compared with type I collagen is reduced and changes in cross-linking occur. It was shown that increased proportion of type I collagen to type III collagen (studies on prolidase deficiency with a possible defect in collagen metabolism) was related with increased prolidase activity. Serum prolidase activity may be interpreted by increased collagen breakdown and turnover. Collagen and proteoglycans found in the structure of joint cartilage. The increased prolidase activity in patients with hip dysplasia implicates the increased collagen breakdown and re-synthesis (collagen turnover) [27, 28].

We found positive correlation between serum prolidase activity and classification criteria for DDH. It appears that prolidase level was affected with the severity of disease. We also did not found any correlation between mean prolidase activity and age, family history, disease duration.

Several limitations of this study should be considered. The present work has a cross-sectional study design and it has been undertaken on the small number of subjects. A larger population studies are needed to elucidate whether these alterations are consistent and have clinical relevance. Another potential limitation of this study was the lack of different age group patients with DDH. Future research on the different clinical outcomes of DDH in the different age group patients will further understanding of the etiopathogenesis of the disease and potentially provide a rationale for targeted treatment interventions.

We thought that collagen metabolism, prolidase activity and DDH might be associated with each other which may be interpreted as evidence of increased collagen turnover in DDH. To the best of our knowledge, there is no data concerning the serum prolidase activity in subjects with DDH. We think that more studies with larger case groups are needed to investigate serum prolidase activity and its relation with collagen metabolism in the future. Initiation and progression of DDH remain to be evaluated with further studies.
